# Lactopeptine

**Published:** 1881-07

**Authors:** 


LACTOPEPTINE.—This preparation, which is composed of pepsin pancreatine and diastase with lactic and hydrochloric acid, has in many cases marked advantage over pepsin alone. We have found it an admirable remedy for the ailments to which children are especially subject in hot weather and which largely depend upon impaired digestion.

